# Trends in prescribing and outcomes in obese versus non-obese patients receiving rivaroxaban therapy: an observational study using real-world data

**DOI:** 10.1007/s00228-023-03572-7

**Published:** 2023-10-10

**Authors:** Majdoleen Alalawneh, Ousama Rachid, Ibtihal Abdallah, Ahmed Mahfouz, Hazem Elewa, Mohammed Ibn-Mas‘ud Danjuma, Asmaa Ezzeldin Mohamed, Ahmed Awaisu

**Affiliations:** 1 College of Pharmacy, QU Health, Qatar University, P.O. Box 2713, Doha, Qatar; 2 Clinical Pharmacy Services, Hamad General Hospital, Hamad Medical Corporation, P.O. Box 3050, Doha, Qatar; 3 Pharmacy Department, Heart Hospital, Hamad Medical Corporation, P.O. Box 3050, Doha, Qatar; 4 Department of Internal Medicine, Hamad General Hospital, Hamad Medical Corporation, P.O. Box 3050, Doha, Qatar; 5 College of Medicine, QU Health, Qatar University, P.O. Box 2713, Doha, Qatar

**Keywords:** Rivaroxaban, Direct oral anticoagulants, Body mass index, Obese, Clinical outcomes

## Abstract

**Purpose:**

To investigate real-world prescribing trends and clinical outcomes based on body mass index (BMI) categorization in patients who received rivaroxaban therapy.

**Methods:**

This was a retrospective cohort study involving all patients who received rivaroxaban therapy across all Hamad Medical Corporation (HMC) hospitals from 2015 to 2020.

**Results:**

The number of patients initiated on rivaroxaban therapy significantly increased from 152 (3.3%) in 2015 to 1342 (28.9%) in 2020 (*p* <0.001). Within BMI categories, a similar increasing trend was observed in underweight, normal, and overweight patients, while from 2018 to 2020, there was a decreasing trend in rivaroxaban prescribing in all obese classes. The prevalence rate of all-cause mortality differed significantly between the BMI groups, with the highest mortality being among morbidly obese patients (BMI ≥ 40 kg/m^2^) (*p*< 0.001). On the other hand, no significant differences were found between the BMI groups in terms of bleeding, pulmonary embolism, deep vein thrombosis and stroke incidences. Multivariate logistic regression analyses showed that the likelihood of all-cause mortality was significantly higher in overweight and all categories of obese patients compared to underweight patients: overweight (OR: 5.3, 95% CI: 2.3–11.9, *p*< 0.001); obese class 1 (OR: 5.4, 95% CI: 2.3 – 12.2, *p*< 0.001); obese class 2 (OR: 6.5, 95% CI: 2.7 – 15.6, *p*< 0.001); and obese class 3 (OR: 3.7, 95% CI: 1.6 – 8.7, *p* = 0.003).

**Conclusions:**

Rivaroxaban prescribing has significantly increased over the years across general population, with a noticeable decline in obese population during the last few years (from 2018 onwards). Furthermore, an appreciable association was evident between all-cause mortality and BMI of these patients.

## Introduction

Thromboembolic disorders constitute a substantial burden to the global healthcare system due to their high prevalence and severe complications [1]. According to estimates, venous thromboembolism (VTE) was the third most prevalent cardiovascular condition [1, 2], with an annual incidence of 1 million individuals in the United States (US) and more than 700,000 individuals in Europe [3]. On the other hand, the global prevalence of atrial fibrillation (AF) was estimated to be 37.6 billion cases (0.51% of world population), and this prevalence has increased in the last two decades by 33% [4]. Anticoagulants are the cornerstone in the treatment and prevention of several thromboembolic disorders, including VTE, and stroke prevention in non-valvular atrial fibrillation (NVAF) [2, 5, 6]. In recent years, direct oral anticoagulants (DOACs) have attracted a considerable interest due to their proven effectiveness, safety, and convenience of use for patients [7–11]. DOACs have become the first line treatment for these disorders [12].

Rivaroxaban is the first direct oral anti-Xa agent approved by the European Medicine Agency and the US’s Food and Drug Administration [13]. The estimated number of rivaroxaban prescriptions in the US was about 9 million in 2019, with rivaroxaban ranked 91^st^ among the top 300 drug list in the US [14]. Rivaroxaban is currently approved for a number of clinical indications, including stroke prevention in NVAF, treatment of deep vein thrombosis (DVT) and pulmonary embolism (PE), primary prevention of VTE post-hip and knee replacement, secondary prevention of VTE recurrence, and risk reduction of major adverse cardiovascular events (MACE) in myocardial infarction, coronary artery disease and peripheral arterial disease [15–17].

Although rivaroxaban has received much attention in recent years due to multiple advantages [15, 18, 19], there is conflicting evidence in terms of its exposure and pharmacokinetics in patients with large body weight, and thus clinical efficacy and safety, particularly when long-term standard doses are used [12, 20, 21]. This may be related to the limited pharmacokinetics /pharmacodynamics (PK/PD) data available for rivaroxaban in extreme weight patients, which makes it very challenging to have conclusive and consistent recommendations regarding its use in clinical practice [22]. In addition, since rivaroxaban is a relatively novel anticoagulant, the trend of its prescribing and associated clinical outcomes in relation to body weight and body mass index (BMI) have not been widely investigated, especially in the Middle East and North Africa (MENA) region. For example, one study was conducted to explore the trends of prescribing three oral anticoagulants—including rivaroxaban—from 2011 to 2015 in Qatar [23]; however, the study did not consider the body weight (body mass index) as a factor affecting the prescribing trends. To the best of the authors' knowledge, there has been no investigations conducted locally or internationally on the prescribing trends of rivaroxaban based on BMI categorization. The aim of this study was to explore the prescribing trends and clinical outcomes of rivaroxaban therapy based on BMI categorization using real-world data in Qatar.

## Method

### Study design and setting

This was a retrospective cohort study involving patients of different BMI categories who were prescribed rivaroxaban therapy at Hamad Medical Corporation (HMC) from 2015 to 2020. HMC is the principal public healthcare provider in the State of Qatar and consists of 12 general and specialized hospitals. HMC facilities deliver medical services to more than 80% of the population in Qatar [24]. The study data were obtained from all HMC facilities where patients received rivaroxaban therapy.

### Study subjects

All adult patients (aged ≥ 18 years) who were prescribed rivaroxaban therapy, either as in-patients or outpatients from 1 January 2015 to 31 December 2020, were eligible for inclusion in the study. Patients were stratified by two dimensions: BMI and year of rivaroxaban therapy prescription. For BMI stratification, patients were classified into six categories: under-weight (<18.5 kg/m^2^), normal weight (18.5 to 24.99 kg/m^2^), overweight (25 to 29.99 kg/m^2^), obese class 1 (30 to 34.99 kg/m^2^), obese class 2 (BMI of 35 to 39.99 kg/m^2^), and obese class 3 (≥ 40 kg/m^2^). Per calendar year, patients who had multiple refill prescriptions were counted only once.

### Sample size and sampling technique

For the study sample analyses, universal sampling (i.e. whole population sampling) was used. Thus, all eligible patients (adult patients at HMC who received rivaroxaban therapy from 1 January 2015 to 31 December 2020) were included in the study. Consequently, sample size determination and sampling technique were not warranted in this study.

### Data collection procedures

Data were collected from 5 August 2022 to 9 February 2023. The following data were extracted from HMC’s electronic medical record system, CERNER: patient demographics, baseline clinical characteristics, rivaroxaban-related data (i.e. clinical indication for rivaroxaban use, rivaroxaban dosage regimen, rivaroxaban starting and discontinuation/switching dates), and clinical outcomes of interest (i.e. bleeding, occurrence/recurrence of DVT, PE, stroke, and all-cause mortality). The data were collected by reviewing all prescription records of rivaroxaban and associated clinical notes documented during hospitalization, outpatient clinic visits, or emergency visits to any HMC facility, since all facilities within HMC have an integrated electronic system (i.e. CERNER). All relevant data were manually extracted using a pre-tested data collection form. All missing data were indicated in the results section and relevant tables.

### Outcomes measures

The study’s outcome measures include the following: the number of annual rivaroxaban prescriptions from 2015 to 2020; major/minor bleeding events according to International Society on Thrombosis and Haemostasis (ISTH bleeding) criteria [25, 26]; thrombotic events (stroke, DVT and PE); all-cause mortality. Each patients was retrospectively followed for at least 1 year after initiation of rivaroxaban therapy, or until mortality occurred. Furthermore, retrospective follow-up was continued until determination of lost to follow-up, or until the end of the study (i.e. end of data collection from the electronic system in February 2023). All outcomes were calculated for the total study sample and compared according to the different BMI groups.

### Study covariates

The results of safety and effectiveness outcomes of rivaroxaban therapy based on BMI categorization were adjusted for clinically relevant demographic-related, disease-related and medication-related variables that were associated with thromboembolic diseases, including: gender, nationality of origin, age, BMI, smoking status, number of co-medications, diabetes, hypertension, dyslipidemia, coronary artery disease (CAD), anemia, liver disease, kidney disease, and duration on rivaroxaban therapy [27–32].

### Statistical analysis

Data analyses were performed using IBM Statistical Package for Social Sciences (IBM SPSS Statistics for Windows) version 28 (IBM Corp, Armonk, NY, USA). Continuous variables were expressed as mean ± SD, while categorical variables were expressed as proportions and percentages. Descriptive statistics were applied to explore the trends of annual rivaroxaban prescribing and across the BMI subgroups. The rivaroxaban prescribing trends between BMI groups were compared using Chi-square test. Similarly, clinical outcomes (bleeding, DVT, PE, stroke and all-cause mortality) were compared between BMI groups using Chi-square test.

Multivariate and univariate analyses were conducted to examine the effect of BMI on outcomes of therapy (bleeding, DVT, PE, stroke, and all-cause mortality incidences), while controlling for predetermined confounding variables (gender, nationality, age, BMI, smoking status, number of co-medications, diabetes, hypertension, hyperlipidaemia, coronary artery diseases, anaemia, liver disease, kidney disease, duration on rivaroxaban therapy). All the study variables (gender, nationality, age, BMI, smoking status, number of co-medications, diabetes, hypertension, hyperlipidaemia, coronary artery diseases, anaemia, liver disease, kidney disease, duration on rivaroxaban therapy) were first tested for association with clinical outcomes using univariate analysis. Variables with *p*-value of less than 0.2 were included into multivariate logistic regression models to investigate the predictors of the study outcomes (bleeding, DVT incidence, PE incidence, stroke incidence, and all-cause mortality). For all analyses, *p*-value < 0.05 was considered statistically significant.

## Results

### Subjects’ selection and baseline characteristics

From 1 January 2015 to 31 December 2020, 4663 electronic medical records of patients who received rivaroxaban therapy were identified. Twenty-five records were for patients below 18 years, and thus were excluded from the study. The remaining 4638 patients’ records were included in the analyses. The mean ± SD age and BMI for the study cohort were 51.84 ± 16.8 years and 30.56 ± 7.4 kg/m^2^, respectively. The majority of the study subjects were male (60%) and non-smokers (90.9%).

Stratifying the study subjects by BMI, the overweight group (BMI 25 – 29.99 kg/m^2^) had the highest number of patients [1411 patients (32.2%)], followed by obese class 1 category (BMI 30 – 34.99 kg/m^2^) [1098 patients (25%)]. The rest of the groups were shown in Table 1. Obesity classes 1, 2, and 3 were found to be most common among non-Qatari Arab populations (42.9%, 48.0%, and 52.2%, respectively) compared to Qatari and non-Arab populations (*p*<0.001). For the baseline clinical characteristics, diabetes, hypertension, dyslipidemia, and CAD were highly prevalent in the studied population with proportions of 40.1%, 33%, 15.8%, and 11.4%, respectively; with a significant increase in the proportions of patients with these comorbidities as the BMI increases (*p* <0.001). The mean ± SD number of co-medications per patient was 2.3 ± 2.1, with a significant increase in this number as the BMI increases (*p*<0.001). Table 1 presents details on other demographic and baseline clinical characteristics for the overall study subjects based on BMI categories.

**Table 1:** Demographics and baseline clinical characteristics of patients on rivaroxaban from 2015 to 2020 according to BMI classification (n = 4638)

		**BMI (Kg/m**^**2**^**)**						
**Parameter**	**Total** **(n = 4638)**	**<18.5** **(n = 64)**	**18.5 to 24.99** **(n = 886)**	**25 to 29.99** **(n = 1411)**	**30 to 34.99** **(n = 1098)**	**35 to 39.99** **(n = 535)**	** ≥ 40** **(n = 391)**	***p*-value**
Gender, n (%)								<0.001^a^
Male	2783 (60)	44 (68.8)	625 (70.5)	972 (68.9)	642 (58.5)	221 (41.3)	114 (29.2)	
Female	1855 (40)	20 (31.2)	261 (29.5)	439 (31.1)	456 (41.5)	314 (58.7)	277 (70.8)	
Age (years), n (%)								<0.001^a^
18 to <55	2566 (55.3)	38 (59.4)	539 (60.8)	832 (59)	603 (54.9)	230 (43)	150 (38.4)	
55–75	1665 (35.9)	14 (21.9)	242 (27.3)	456 (32.3)	414 (37.7)	260 (48.6)	213 (54.5)	
>75	407 (8.8)	12 (18.7)	105 (11.9)	123 (8.7)	81 (7.4)	45 (8.4)	28 (7.2)	
Nationality, n (%)								<0.001^a^
Qatari Arab	1105 (23.8)	22 (34.4)	164 (18.6)	277 (19.6)	285 (26)	181 (33.8)	145 (37.1)	
Non-Qatari Arab	1790 (38.6)	15 (23.4)	269 (30.3)	489 (34.7)	471 (42.9)	257 (48.0)	204 (52.2)	
Non-Arab	1743 (37.6)	27 (42.2)	453 (51.1)	645 (45.7)	342 (31.1)	97 (18.1)	42 (10.7)	
Smoking history, n (%)								0.003^a^
Smokers	391 (8.4)	4 (6.3)	94 (10.6)	138 (9.8)	92 (8.4)	38 (7.1)	20 (5.1)	
Non-smokers	4214 (90.9)	60 (93.7)	788 (88.9)	1263 (89.5)	996 (90.7)	492 (92.0)	369 (94.4)	
Ex-smokers	33 (0.7)	0 (0)	4 (0.5)	10 (0.7)	10 (0.9)	5 (0.9)	2 (0.5)	
Heart rate (bpm), mean (SD)	76.06 (10.99)	76.7 (9.8)	75.9 (10.9)	75.8 (11.2)	76.1 (11.2)	75.9 (10.6)	76.6 (10.3)	0.843^a^
SBP (mmHg), mean (SD)	126.63 (15.76)	122 (18.7)	124.3 (15.8)	126.2 (15.7)	127.5 (15.3)	127.9 (15.9)	128.9 (15.9)	<0.001^a^
DBP (mmHg), mean (SD)	76.04 (9.60)	74.4 (9.2)	74.7 (9.2)	76 (9.4)	76.3 (9.5)	76.2 (10.1)	76.9 (10.4)	<0.001^a^
ALT (U/L), mean (SD)	27.90 (37.70)	27.2 (27.6)	31.5 (71.6)	28.7 (25.7)	27.7 (23)	24.4 (19)	23.3 (19.7)	<0.001^a^
AST (U/L), mean (SD)	25.30 (28.00)	31 (20.8)	29.4 (56.5)	25.6 (15.4)	24.6 (16.7)	22 (10.7)	21.9 (13.2)	<0.001^a^
SCr. (μmol/L), mean (SD)	75.65 (29.88)	74.8 (29.6)	75.1 (36.4)	77.61 (30.9)	75.5 (28.4)	73.8 (23.7)	72.8 (24.6)	<0.001^a^
Diabetes mellitus, n (%)	1858 (40.1)	17 (26.6)	293 (33.1)	545 (38.6)	458 (41.7)	273 (51.0)	216 (55.2)	<0.001^b^
Hypertension, n (%)	1533 (33.0)	18 (28.1)	218 (24.6)	428 (30.3)	405 (36.9)	240 (44.9)	191 (48.8)	<0.001^b^
Dyslipidemia, n (%)	773 (15.8)	6 (9.4)	110 (12.4)	210 (14.9)	211 (19.2)	110 (20.6)	111 (28.4)	<0.001^b^
CAD, n (%)	528 (11.4)	5 (7.8)	97 (10.9)	155 (11.0)	139 (12.7)	64 (12.0)	60 (15.3)	0.158^b^
Kidney disease, n (%)	411 (8.9)	6 (9.4)	69 (7.8)	128 (9.1)	99 (9.0)	57 (10.7)	45 (11.5)	0.303^b^
Liver disease, n (%)	94 (2.0)	2 (3.1)	14 (1.6)	22 (1.6)	26 (2.4)	15 (2.8)	15 (3.8)	0.059^b^
Anemia, n (%)	266 (5.7)	3 (4.7)	42 (4.7)	85 (6.0)	64 (5.8)	29 (5.4)	37 (9.5)	0.043^b^
No. of co-medications per patient, mean (SD)	2.3 (2.1)	2.4 (2.1)	2.3 (2.1)	2.5 (2.1)	2.7 (2.1)	3.1 (2.1)	3.4 (2.0)	< 0.001^a^
Co-medications, n (%)								< 0.001^b^
0	1002 (21.6)	12 (18.8)	251 (28.3)	320 (22.7)	208 (18.9)	71 (13.3)	36 (9.2)	
1 to 4	2640 (56.9)	38 (59.4)	477 (53.8)	809 (57.3)	637 (58.0)	322 (60.2)	232 (59.3)	
5 to 10	996 (21.5)	14 (21.8)	158 (17.8)	282 (20.0)	253 (23.0)	142 (26.5)	123 (31.4)	

Total number of patients with known BMI is 4385 as some BMI data were missing from patients’ records

*SBP* Systolic blood pressure, *DBP* Diastolic blood pressure, *ALT* alanine aminotransferase, *AST* aspartate aminotransferase, *SCr* Serum creatinine, *CAD* coronary artery disease

^a^Kruskal Wallis test

^b^chi-square test

### Rivaroxaban prescribing trends among patients with thromboembolic diseases

**Fig. 1:**
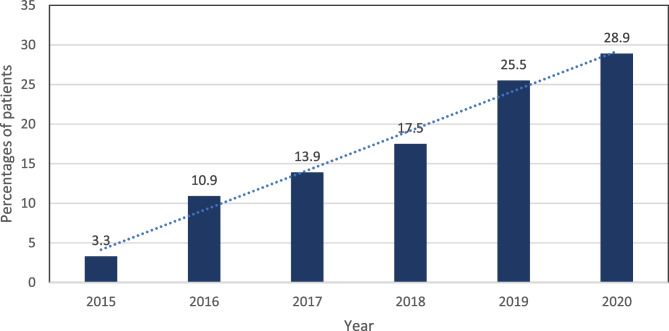
Trend of rivaroxaban utilization across the years from 2015 to 2020 (n = 4638). This figure is Microsoft Excel-generated

There was an increasing trend in rivaroxaban prescribing across the study period for the entire study cohort: 152 patients (3.3%) in 2015, 505 patients (10.9%) in 2016, 646 patients (13.9%) in 2017, 810 patients (17.5%) in 2018, 1183 patients (25.5%) in 2019, and 1342 patients (28.9%) in 2020 (*p* <0.001) (Fig. 1).

**Fig. 2:**
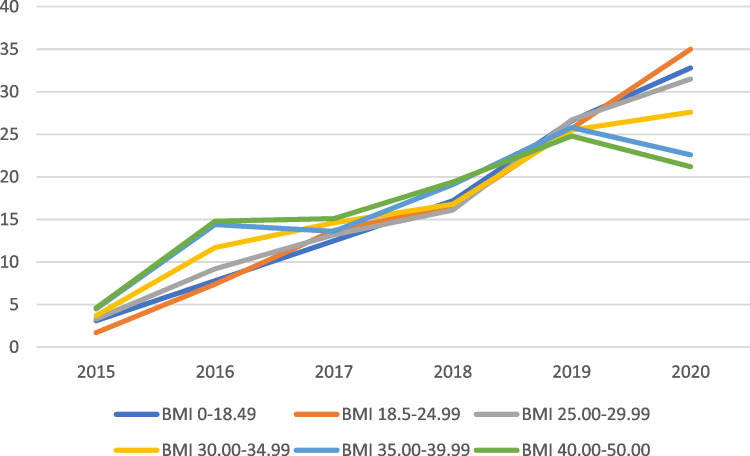
Percentages of patients on rivaroxaban therapy over the study period according to their BMI classification. This figure is Microsoft Excel-generated

Interestingly, the increasing trend in rivaroxaban prescriptions was consistent among the lower BMI categories (underweight, normal, overweight), but not in the obese categories where trends started to decline from 2018 to 2020, i. e., declining percentages of 5%, 31%, and 36% were found in obese class 1, 2 and 3, respectively. Table 2 and Fig. 2 demonstrate the trend in rivaroxaban prescription based on BMI categorization across the study years.

**Table 2:** Number of patients on rivaroxaban therapy over the study period according to their BMI classification (n = 4385)*

	**BMI (Kg/m**^**2**^**)**					
	**<18.5**	**18.5–24.99**	**25.00–29.99**	**30.00–34.99**	**35.00–39.99**	** ≥ 40**
2015 (n = 146)	2 (1.4)	15 (10.3)	46 (31.5)	41 (28.1)	24 (16.4)	18 (12.3)
2016 (n = 465)	5 (1.1)	66 (14.2)	130 (28.0)	129 (27.7)	77 (16.6)	58 (12.5)
2017 (n = 607)	8 (1.3)	121 (19.9)	186 (30.6)	160 (26.4)	73 (12.0)	59 (9.7)
2018 (n = 747)	11 (1.5)	146 (19.5)	227 (30.4)	185 (24.8)	102 (13.7)	76 (10.2)
2019 (n = 1137)	17 (1.5)	228 (20.1)	377 (33.2)	280 (24.6)	138 (12.1)	97 (8.5)
2020 (n = 1283)	21 (1.6)	310 (24.2)	445 (34.7)	303 (23.6)	121(9.4)	83 (6.5)

*Total number of patients with known BMI is 4385 as some BMI data were missing from patients’ records

### Rivaroxaban-related characteristics and clinical outcomes according to body mass index

Table 3 indicates the rivaroxaban dosing regimens and their distribution across the BMI-classified groups. the findings suggest that 20mg rivaroxaban once daily was the most common dosing regimen across the study period [2866 patients, (61.9%)], followed by 10mg rivaroxaban once daily [1310 patients, (28.3%)]. In addition, the prescribing of rivaroxaban 20mg once daily was significantly higher as the BMI of patients increases [i.e., 50% among the underweight, 56.9% among normal weight, 59.5% among overweight, 65.1% among obese class 1, 63.9% among obese class 2, and 65.9% among obese class 3 (*p*<0.001)]. The duration of rivaroxaban therapy significantly increases as the BMI increases (*p*<0.001) (Table 3). The most common clinical indication for the use of rivaroxaban was NVAF (1351 patients, 29.1%), followed by DVT (402 patients, 8.7%). The mean follow-up period for study sample was 1.8 years.

**Table 3:** Rivaroxaban dosing regimen within the study sample according to body mass index (n = 4633)^a^

**BMI (Kg/m**^**2**^**)**								
**Dosing regimen, n (%)**	**Total** (n = 4633)^a^	**<18.5** **(n = 64)**	**18.5 to 24.99 (n = 885)**	**25 to 29.99 (n = 1409)**	**30 to 34.99 (n = 1097)**	**35 to 39.99 (n = 535)**	** ≥ 40** **(n = 390)**	***p*-value**
2.5 mg twice a day	1 (0.02)	0 (0.0)	0 (0.0)	0 (0.0)	0 (0.0)	0 (0.0)	1 (0.3)	<0.001^b^
10 mg once a day	1310 (28.3)	22 (34.4)	297 (33.6)	416 (29.5)	299 (27.2)	137 (25.6)	87 (22.3)	
15 mg once a day	456 (9.8)	10 (15.6)	84 (9.5)	154 (11.0)	84 (7.7)	56 (10.5)	45 (11.5)	
20 mg once a day	2866 (61.9)	32 (50.0)	504 (56.9)	839 (59.5)	714 (65.1)	342 (63.9)	257 (65.9)	
Duration on rivaroxaban (days), mean (SD)	662.1 ± 650.9	478.1 ± 528.2	525 ± 544.2	621.7 ± 627.9	694 ± 683.2	817.8 ± 714.3	852.4 ± 732.1	<0.001^c^

^a^Some data were missing (the total number of patients with known dosing regimen is 4633)

^b^Chi-square test

^c^Kruskal Wallis

For the total study sample (4638 patients), 1156 outcome events were identified during the follow-up. After adjustment for follow-up duration and number of patients, the incidence rates of clinical outcome events in the study sample were 1.01, 4.89, 4.44, 0.92, 2.38, and 4.14 per 100-person year for major bleeding, minor bleeding, DVT, PE, stroke, and all-cause mortality, respectively. Interestingly, there was a significant difference in all-cause mortality across the BMI groups (*p* <0.001), but not minor bleeding (*p* = 0.087), major bleeding (*p* = 0.987), DVT recurrence (*p* = 0.956), PE recurrence (*p* = 0.170), or stroke (*p* = 0.886). Although the differences were not statistically significant regarding minor bleeding and DVT, the highest incidences of these cardiovascular events occurred among morbidly obese patients compared to the other BMI groups (11.8% and 6.9% respectively). Table 4 represents more details about the clinical indications and the clinical outcomes of rivaroxaban therapy in the study sample.

**Table 4:** Clinical indication and outcomes of rivaroxaban therapy within the study sample

**BMI (Kg/m**^**2**^)								
**n (%)**	**Total** **(n = 4638)**	**<18.5 (n = 64)**	**18.5–24.99** **(n = 886)**	**25.00–29.99** **(n = 1411)**	**30.00–34.99** **(n = 1098)**	**35.00–39.99** **(n = 535)**	** ≥ 40** **(n = 391)**	***p*-value**^**b**^
**Clinical indications**^**a**^								<0.001
AF	1351 (29.1)	21 (32.8)	207 (23.4)	396 (28.1)	350 (31.9)	174 (32.5)	167 (42.7)	
PE	192 (4.1)	1 (1.6)	37 (4.2)	49 (3.5)	41 (3.7)	28 (5.2)	28 (7.2)	
DVT	402 (8.7)	3 (4.7)	60 (6.8)	120 (8.5)	107 (9.7)	54 (10.1)	23 (5.9)	
DVT + PE	69 (1.5)	0 (0.0)	8 (0.9)	15 (1.1)	19 (1.7)	13 (2.4)	10 (2.6)	
Hip and knee replacement	39 (0.8)	0 (0.0)	9 (1.0)	9 (0.6)	14 (1.3)	4 (0.7)	2 (0.5)	
Others^c^	140 (3.0)	3 (4.7)	25 (2.8)	53 (3.8)	31 (2.8)	14 (2.6)	10 (2.6)	
Unspecified	2445 (52.7)	36 (56.3)	540 (60.9)	769 (54.5)	536 (48.8)	248 (46.4)	151 (38.6)	
**Clinical outcomes**								
Bleeding								
Major	72 (1.6)	1 (1.6)	21 (2.4)	21 (1.5)	18 (1.6)	9 (1.7)	8 (2.0)	0.987
Minor	372 (8.0)	5 (7.8)	61 (6.9)	107 (7.6)	96 (8.7)	50 (9.3)	45 (11.8)	0.087
DVT	270 (5.9)	3 (4.7)	50 (5.6)	82 (5.8)	64 (5.8)	30 (5.6)	27 (6.9)	0.956
PE	41 (0.9)	0 (0.0)	3 (0.3)	17 (1.2)	13 (1.2)	3 (0.6)	2 (0.5)	0.170
Stroke	138 (3.0)	1 (1.6)	29 (3.3)	46 (3.3)	31 (2.8)	13 (2.4)	12 (3.1)	0.886
All-cause mortality	263 (5.7)	12 (18.8)	71 (8.0)	63 (4.5)	52 (4.7)	27 (5.0)	33 (8.4)	< 0.001

*AF* Atrial fibrillation, *PE* Pulmonary embolism, *DVT* Deep vein thrombosis

^a^Some clinical indication data were missing (total number of patients with known clinical indication is 2193)

^b^Chi-square test

^c^Include catheter related thrombosis, case of antiphospholipid syndrome with right big toe ischemia, isolated great saphenous vein thrombosis, cerebral vein thrombosis, protein C deficiency, mesenteric or portal vein thrombosis, sagittal vein thrombosis, rectal clots with ulcer, portal vein thrombosis, protein S deficiency, splenic vein thrombosis, superficial venous thrombosis, brachial artery thrombosis, history of right ovarian vein thrombosis, rheumatic Heart Disease, mild to moderate mitral stenosis, intracardiac thrombosis

Multivariate logistic regression analyses showed that all-cause mortality was higher in different categories of obese patients as follows: being obese class 1 (OR: 5.4, 95% CI: 2.3–12.2, *p* <0.001); being obese class 2 (OR: 6.5, 95% CI: 2.7–15.6, *p* <0.001); being obese class 3 (OR: 3.7, 95% CI: 1.6–8.7, *p* = 0.003) when compared to underweight BMI group mortality rates. Regression analysis did not show significance for DVT recurrence and stroke with any variables. More details about the regression analyses results are shown in Table 5.

**Table 5:** Multiple logistic regression for the predictors of clinical outcomes among patients receiving rivaroxaban therapy

Clinical outcomes	*p*-value	Odd ratios	95% CI for population odd ratio
**Major bleeding**^**a**^			
Female gender	0.015	1.9	1.138 – 3.323
Number of co-medications	< 0.001	1.301	1.120 – 1.510
Diabetes	0.044	1.835	1.017 – 3.310
Hyperlipidaemias	0.037	1.989	1.043 – 3.796
Liver disease	0.025	0.345	0.136 – 0.876
**Minor bleeding**^**a**^			
Female gender	< 0.001	1.569	1.223 – 2.013
Nationality (being Qatari)	0.003	1.622	1.178 – 2.233
HTN	< 0.001	0.571	0.428 – 0.763
Anaemia	< 0.001	0.486	0.343 – 0.691
Duration on rivaroxaban	< 0.001	1.00	1.00 – 1.00
**PE**			
Age	0.009	1.035	1.009 – 1.062
**Mortality**			
Nationality (being Arabs, non-Qatari)	0.034	1.391	1.026 – 1.886
Nationality (being non-Arabs)	< 0.001	2.166	1.445 – 3.249
Age	< 0.001	0.944	0.934 – 0.954
BMI (obese class 1)	< 0.001	5.354	2.352 – 12.191
BMI (obese class 2)	< 0.001	6.536	2.739 – 15.600
BMI (obese class 3)	0.003	3.700	1.567 – 8.737
Number of medications	0.002	0.879	0.811 – 0.953
Diabetes	0.010	1.537	1.008 – 2.132
Hyperlipidaemia	< 0.001	0.471	0.334 – 0.664
Anaemia	0.013	1.741	1.124 – 2.698
Kidney disease	0.025	1.488	1.051 – 2.107

^a^Reference group is NO bleeding; Only variables which have statistically significant *p* values in the multiple logistic regression model are displayed in this table

## Discussion

This study investigates the trends of rivaroxaban prescription as well as associated clinical outcomes with a focus on BMI categories. Overall, the study findings showed that rivaroxaban prescription has increased from 3.3% in 2015 to 28.9% in 2020 (i.e. more than eightfold increase across the 5-year time span), which substantiates previous findings in the published literature [23, 33, 34]. Notably, an overall increase of rivaroxaban prescription was also reported among all BMI groups between 2015 and 2020 (i.e., 9.6, 19.6, 8.5, 6.5, 4, and 3.6 folds among underweight, normal weight, overweight, obese class 1, obese class 2, and obese class 3, respectively) (Fig. 2). This trend in rivaroxaban prescription reported in our study is consistent with the global rivaroxaban prescribing trends, the rank of which has increased from 132 in 2015 to 86 in 2020 [14]. The rank refers to the frequency that a given medication is prescribed within a calendar year compared to all other medications.

It is noteworthy that the increasing trend in the prescription of rivaroxaban remained steady in the lower BMI categories (underweight, normal weight, and overweight patients), while the utilization began to decline in all the obese categories in the last 2 years of the retrospective data analysis (Fig. 2). This distinction in the trend of rivaroxaban prescribing in obese vs. non-obese populations may reflect clinicians’ hesitance in prescribing rivaroxaban for obese population in light of the emerging and conflicting evidence and/or recommendations in recent years [35–39]. Such key recommendations were reported in the ISTH position statement "we suggest that DOACs should not be used in patients with a BMI of > 40 kg/m^2^ or a weight of > 120 kg, because there are limited clinical data available for patients at the extreme of weight, and the available PK/PD evidence suggests that decreased drug exposures, reduced peak concentrations and shorter half‐lives occur with increasing weight, which raises concerns about underdosing in the population at the extreme of weight", which emphasizes the usefulness of exploring such prescription trends of rivaroxaban in real-world practice [40–42].

With regard to clinical outcomes, our findings suggest that morbidly obese patients had significantly higher numbers of all-cause mortality (8.4%) compared to other BMI groups (except underweight category). The all-cause mortality rate reported in the morbidly obese patients in this study was comparatively higher than the 6.2% and 5.6% reported in previous studies [43, 44], and fairly close to the 7.1% all-cause mortality reported in another study [45]. It should be highlighted that the higher mortality rate observed in underweight patients (18.8%) may be attributed to the large proportion of elderly patients (≥ 75 years old) in this group [46]. Conversely, DVT incidence was not significantly different between BMI groups; the finding that is in agreement with one prior study [47], and in contradiction with some earlier studies [48, 49] that have found a remarkable association between BMI and DVT during anticoagulation, and this might be due to the differences in study settings, designs, time span covered by the studies, and other confounding factors. On the other hand, our DVT results are almost indistinguishable from several previous studies which have raised the concern about thromboembolic events recurrence and the subclinical effect during rivaroxaban anticoagulation therapy among obese patients [21, 42]. For minor bleeding incidence in morbidly obese patients, the reported 11.8% in this study was considerably higher than what was reported in earlier studies [9, 44].

Multivariate logistic regression results showed that hypertension and number of medications have affected the bleeding outcome in the current study, the findings which were consistent with previous studies [50–52]. On the other hand, female gender effect on bleeding was not confirmed by previous studies in the literature as both genders were having the same bleeding risk upon rivaroxaban use [53]. Although our results that showed the effect of diabetes on bleeding outcome for patients on rivaroxaban were not found in earlier studies, the hemorheological changes that diabetes cause in general population (in the context of anticoagulation) have been extensively discussed in the literature [54–56]. Similarly, the effect of liver diseases on bleeding is well-known [57, 58]; however, it was not investigated in the context of anticoagulation. As for the duration of rivaroxaban therapy effect on bleeding, and in accordance with our findings, many studies confirmed the increased risk of bleeding with therapy duration increase for oral anticoagulants [59–63]. Furthermore, our results regarding age as a risk factor for both PE and for all-cause mortality were in line with previous finding in the literature, respectively [64–67]. With regard to the effect of age, diabetes, and kidney disease on all-cause mortality outcome, our study results concur well with the previous studies [68–72].

This study has some limitations, most of which are inherent to all retrospective studies. First is that the obtained results were based on HMC records only, and even though HMC is the principal public healthcare provider in the State of Qatar, it cannot be ruled out that the results would change if the private hospitals and centres were included. Second, it is plausible that the HMC’s electronic medical records have some errors or inaccuracies that are unaccounted for, thereby affecting the analyses. Third, including data from 2021 and 2022 records would be advisable, as it would provide a more complete picture of rivaroxaban prescription trends. However, this two-year gap was not included to allow for outcomes capturing among patients (i.e. clinical outcomes for rivaroxaban use were captured until December 2021). Lastly, this study did not assess the effect of potential interacting co-medications, which could possibly affect the current study findings and their interpretation.

## Conclusion

This study has generated much-needed information regarding the trends in rivaroxaban utilization and clinical outcomes in general population and in different BMI categories. The findings lead us to conclude that the prescribing trends of rivaroxaban has increased significantly over the years and has been observed across different BMI categories. We have obtained comprehensive real-world evidence demonstrating that patients with a morbidly obese BMI receiving rivaroxaban therapy are at a significantly higher risk of all-cause mortality. The findings add substantially to a growing body of literature on rivaroxaban prescribing trends, efficacy, and safety in obese population.

## Data Availability

The data related to this study are available on request from the corresponding author. However, the dataset are not publicly available due to privacy and ethical restrictions.
